# Parkinson’s Disease Diagnosis and Severity Assessment Using Ground Reaction Forces and Neural Networks

**DOI:** 10.3389/fphys.2020.587057

**Published:** 2020-11-09

**Authors:** Srivardhini Veeraragavan, Alpha Agape Gopalai, Darwin Gouwanda, Siti Anom Ahmad

**Affiliations:** ^1^Advanced Engineering Platform, School of Engineering, Monash University Malaysia, Subang Jaya, Malaysia; ^2^Malaysian Research Institute on Ageing, Universiti Putra Malaysia, Selangor, Malaysia

**Keywords:** gait analysis, artificial neural {network (ANN)}, Parkinson’s Disease, machine learning, SMOTE

## Abstract

Gait analysis plays a key role in the diagnosis of Parkinson’s Disease (PD), as patients generally exhibit abnormal gait patterns compared to healthy controls. Current diagnosis and severity assessment procedures entail manual visual examinations of motor tasks, speech, and handwriting, among numerous other tests, which can vary between clinicians based on their expertise and visual observation of gait tasks. Automating gait differentiation procedure can serve as a useful tool in early diagnosis and severity assessment of PD and limits the data collection to solely walking gait. In this research, a holistic, non-intrusive method is proposed to diagnose and assess PD severity in its early and moderate stages by using only Vertical Ground Reaction Force (VGRF). From the VGRF data, gait features are extracted and selected to use as training features for the Artificial Neural Network (ANN) model to diagnose PD using cross validation. If the diagnosis is positive, another ANN model will predict their Hoehn and Yahr (H&Y) score to assess their PD severity using the same VGRF data. PD Diagnosis is achieved with a high accuracy of 97.4% using simple network architecture. Additionally, the results indicate a better performance compared to other complex machine learning models that have been researched previously. Severity Assessment is also performed on the H&Y scale with 87.1% accuracy. The results of this study show that it is plausible to use only VGRF data in diagnosing and assessing early stage Parkinson’s Disease, helping patients manage the symptoms earlier and giving them a better quality of life.

## Introduction

Parkinson’s Disease (PD) is a highly prevalent neuro-degenerative disease that affects more than 10 million people worldwide. While PD usually occurs in adults aged 50 and above, there have been cases of young onsets of this disease, where individuals as young as 18 years old have been diagnosed with PD (Parkinson’s Foundation, 2019). There are five progression stages in PD, where treatment in the early stages (Stages 1 and 2) slows down the onset of the disease, allowing patients to experience a better quality of life (Parkinson’s Foundation, 2019). However, there is no specific test that exists to diagnose PD, and patients will have to rely on a neurologist for a diagnosis.

Neurologists typically base their diagnosis on several factors such as the patients’ medical history, signs and symptoms exhibited, and a neurological and physical examination. Although there are existing scans which may help support neurologists’ in verifying their diagnosis, it is the exhibited symptoms and neurological examination that carries the most weight in the diagnosis. This makes detection of PD in the early stages difficult as the exhibited symptoms are relatively mild and may require several visits to the neurologist before it can be confirmed (Parkinson’s Foundation, 2019). These procedures can be taxing emotionally, financial and in terms of time for both patient and caregiver (Parkinson’s Foundation, 2019).

Common symptoms observed by individuals suffering from PD include postural instability, tremor, slowness in movement and other forms of gait (Parkinson’s Foundation, 2019) due to the deterioration of neurons in the brain. These symptoms start mildly and only escalate with the progression of the disease. Previous studies have investigated the potential of assessing changes in patterns of alteration in gait to aid the diagnosis and quantification of PD (Koozekanani et al., 1987; Salarian et al., 2004; Schlachetzki et al., 2017). These alterations were measured using non-intrusive wearable motion sensors which allow observation of natural day-to-day movements. These movements offer better insight into their individualized gait characteristics. The use of Force-Resistive Sensors (FRS) at the sole of the feet to measure gait events has been studied in the past (Goldberger et al., 2000), and also studies of FRS coupled with gyroscopes and accelerometers (Tadano et al., 2013; Bhosale et al., 2016). Given the significant advancements made in the miniaturization and processing speeds of these sensors, there is great potential in using wearable sensors for early diagnosis of PD.

A common conclusion from past studies of both diagnosis and severity assessment research is that a consistently higher gait cycle duration is observed in PD patients (Koozekanani et al., 1987; Salarian et al., 2004; Tadano et al., 2013; Bhosale et al., 2016; Schlachetzki et al., 2017). Recognizing this, we seek to investigate the possibility of using only wearable sensors to identify PD in its early stages and estimate PD severity. As a proof of concept, we use data previously reported in Goldberger et al. (2000). A key measurement in this dataset is the VGRF measured from the FRSs in the insoles of the feet. Many studies in the past have used this parameter to investigate and quantify gait variability of PD patients (Manap et al., 2011; Abdulhay et al., 2018). However, to the best of our knowledge, no study has explicitly researched on the sole use of gait features for severity assessment, as research in the area of severity assessment primarily focuses on the use of features extracted from speech data (Salarian et al., 2004; Benmalek et al., 2015; Schlachetzki et al., 2017; Grover et al., 2018; Nilashi et al., 2018), in addition to VGRF data. A successful implementation will enable early seamless diagnosis and assessment of PD using only VGRF data.

## Experimental Setup

### Data Description

The database (Goldberger et al., 2000) consists of 93 idiopathic PD patients (58 male and 35 female, Age = 66.3 ± 9.5 years, Height of 1.67 ± 0.084 m and an Weight of 72.4 ± 11.83 kg) and 73 healthy control subjects (40 male and 33 female, Age = 63.7 ± 8.64 years, Height of 1.68 ± 0.085 m and Weight = 72.8 ± 12.26 kg).

**FIGURE 1:**
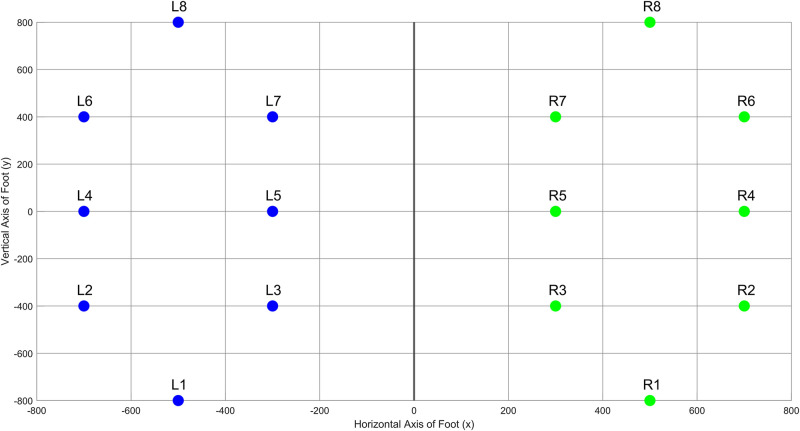
Positioning of the sensors on an arbitrary relative coordinate system on the foot insole.

The database includes the vertical ground reaction force (VGRF) records of subjects as they walked at their usual, self-selected pace for approximately 2 min on level ground. Underneath each foot were 8 sensors (Ultraflex Computer Dyno Graphy, Infotronics Inc.) that measure force (in Newtons) as a function of time. The 8 sensors are arranged on the soles of the feet as follows- 3 sensors each are placed along the inner and outer longitudinal arch, and a sensor each on base of the foot and the heel bone. The approximate coordinates of the sensor locations inside the insole are illustrated in Figure 1, whereby the x and y axes reflect an arbitrary coordinate system (Goldberger et al., 2000) to scale the sensor positions, with the origin in the center between both feet and the person is facing the positive side of the *y* axis. This arbitrary coordinate system is relative to the positions of the sensors, thereby making the sensors inside the insole remain at the same relative coordinate during walking, but the feet are no longer parallel to each other (Goldberger et al., 2000). The output of each of these 16 sensors has been digitized and recorded at 100 samples per second, and the records also include two signals that reflect the sum of the 8 sensor outputs for each foot.

The database also includes qualitative measures of disease severity, including the Hoehn & Yahr (H&Y) staging for subjects suffering from PD. Clinicians use the H&Y score to quantify the level of disability in patients (Parkinson’s Foundation, 2019). A higher H&Y score corresponds to higher disease progression, and thus gait impairment associated with reduced mobility is more prevalent. The H&Y score is a gross assessment of the level of disability through staging, and ranges from stages 0 to 5, where 0 implies no signs of disease and 5 corresponds to a subject being fully impaired or bedridden. All the PD patients are of H&Y stage between 2 and 3 (Average = 2.26 ± 0.34). This implies that the patients in this database are of an early to moderate onset of PD (Goldberger et al., 2000).

### Signal Processing and Feature Extraction

**FIGURE 2:**
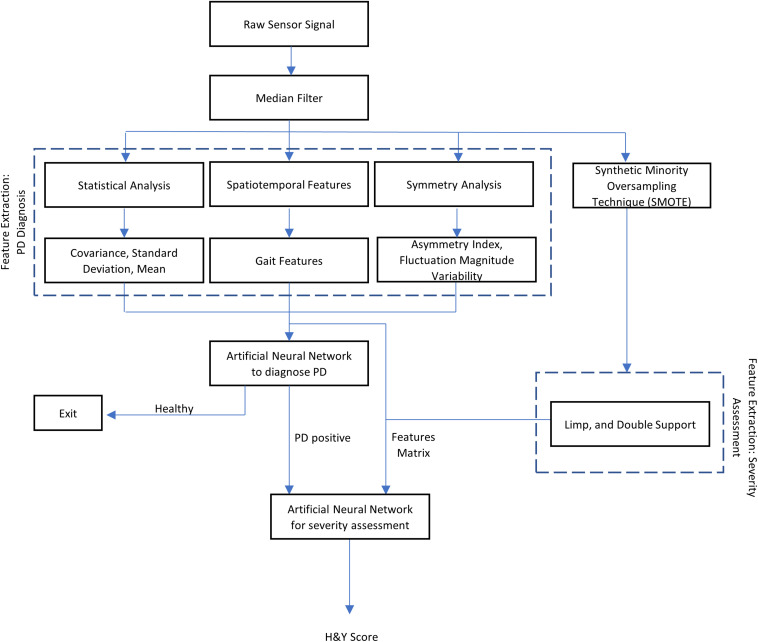
Flowchart of the research methodology used in this study.

All VRGF data were first filtered through a median filter to smoothen the signal and remove outliers (Salarian et al., 2004). This filtered signal is then processed to retrieve useful gait features. The steps to process the data and extract useful features is illustrated in Figure 2.

#### Feature Extraction for PD Diagnosis

**FIGURE 3:**
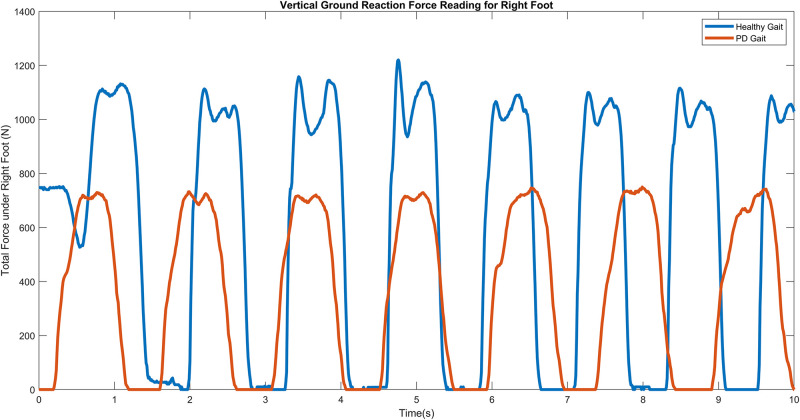
VGRF against time for healthy (Orange) vs. PD (Blue) subjects.

A PD subject generally records approximately 40% longer gait cycle time compared to a healthy subject, with notable lower stride velocity and stance periods, and as the disease progresses, patients may exhibit a single narrow peak force plot (Gaenslen and Daniela, 2010), characterized by a flat foot strike as opposed to a sharp heel strike in control subjects. Severe PD subjects may even exhibit toe-to-heel walking where the toe impacts the ground before the heel or mid-foot (Goetz et al., 2008). Figure 3 illustrates the difference in VGRF reading for a control vs. PD subject.

##### Spatiotemporal features

These features were extracted using the equations tabulated in Table 1, where t represents the start and end indices of the gait cycle events, T represents time, R represents ratio, V represents velocity and i is the corresponding gait cycle iteration. To ensure accuracy of calculation, the summation of the swing and stance times were cross-checked against the stride time.

**TABLE 1:** Gait cycle time events.

**Feature**	**Formula**
Gait cycle/Stride time	GCT (i)= t_*start*_ (i+1)-t_*start*_ (i) (1)
Stance time	T_*stance*_ (i)= t_*end*_ (i)-t_*start*_ (i) (2)
Swing time	T_*swing*_ (i)= t_*start*_ (i+1)-t_*end*_ (i)(3)
Stance ratio	R_*stance*_ (i)= T_*stance*_ (i)/T_*stride*_ (i)(4)
Swing ratio	R_*swing*_ (i) = T_*stride*_ (i)/T_*stance*_ (i) (5)
Swing-stance ratio	R_*swing–stance*_ (i) = T_*swing*_ (i)/T_*stance*_ (i)(6)
Stride length	T_*stride*_ × V_*stride*_ (7)

##### Asymmetry indices

Normalize the value of one side relative to the other, as shown in Eq. 8 which expresses the difference in the stance times of each foot as a fraction of the left stance time. This feature allows easy quantification of inter-individual comparisons (Nadeau, 2014). Using Eq. (8), the Asymmetry Index Ratio was calculated for each gait cycle and averaged over the subjects’ entire walking duration of 2 min to get their individual Asymmetry Index ratio.

(8) $$A\,s\,y\,m\,m\,e\,t\,r\,y\,I\,n\,d\,e\,x\,R\,a\,t\,i\,o=\left|L_{s\,t\,r\,i\,d\,e}\,\left(i\right)-R_{s\,t\,r\,i\,d\,e}\,\left(i\right)\right|/L_{s\,t\,r\,i\,d\,e}\,\left(i\right)$$

##### Statistical analysis

Coefficient of variation (CV), Mean and Standard Deviation (STD) were chosen to assess gait variability in control and PD subjects. This is calculated for Left (L1 to L8) and Right (R1 to R8) feet for both PD and control subjects. Then, the variability in fluctuation magnitude can be computed for each of the eight sensors as shown in Figure 1 which is given by the difference in left and right sensor readings for each sensor as shown in Eq. 9.

(9) F⁢l⁢u⁢c⁢t⁢u⁢a⁢t⁢i⁢o⁢n⁢⁢⁢M⁢a⁢g⁢n⁢i⁢t⁢u⁢d⁢e⁢⁢V⁢a⁢r⁢i⁢a⁢b⁢i⁢l⁢i⁢t⁢y⁢⁢(F⁢M⁢V)=|L⁢i-R⁢i|/Li×100%

where L_*i*_ and R_*i*_ represent the Left or Right *i*^*th*^ sensor, and *i* is the sensor number corresponding to Figure 1. We determined that sensors number 3 and 7 demonstrate the highest variability, as shown in Table 2 and thus were selected as inputs to the classifier.

**TABLE 2:** Highest fluctuation magnitude variability (FMV) exhibited by sensors 3 and 7.

**Sensor**	**1**	**2**	**3**	**4**	**5**	**6**	**7**	**8**
FMV	0.69	0.15	2.45	1.27	2.12	1.71	2.22	0.9

Table 3 summarizes the total list of feature vectors composed of the gait features and their statistical analyses to result in a total of 34 unique features as inputs to the classifier, where n is the unique feature count.

**TABLE 3:** Summary of unique extracted input features.

**Feature**	**n**	**Description**
Primary feature vector	4	A vector consisting of: - Normalized Aggregated Left VGRF - Normalized Aggregated Right VGRF - FMV for sensor 3 - FMV for sensor 7
Stance times	2	Left and Right Stance Times
Stride times	2	Left and Right Stride Times
Swing times	2	Left and Right Swing Times
Swing ratio	2	Left and Right Swing Ratios
Swing ratio	2	Left and Right Stance Ratios
Swing-stance ratio	2	Left and Right Swing-Stance Ratios
Initial contact force	2	Right and Left Maximum Initial Contact Force
Terminal contact force	2	Right and Left Maximum Terminal Contact Force
Statistical covariance	4	Covariance (CoV) of each of the Feature Vector Elements: - CoV(Normalized Aggregated Left VGRF) - CoV(Normalized Aggregated Right VGRF) - CoV(FMV for sensor 3) - CoV(FMV for sensor 7)
Statistical mean	4	Mean of each of the Feature Vector Elements: - mean(Normalized Aggregated Left VGRF) - mean(Normalized Aggregated Right VGRF) - mean(FMV for sensor 3) - mean(FMV for sensor 7)
Standard deviation	4	Mean of each of the Feature Vector Elements: - std(Normalized Aggregated Left VGRF) - std(Normalized Aggregated Right VGRF) - std(FMV for sensor 3) - std(FMV for sensor 7)
Step distance	1	Average Step Distance of the Subjects
Asymmetry index	1	Asymmetry Index of the Stride between left and right feet
**Total Features**	34	

#### SMOTE and Feature Extraction for PD Severity Assessment

During feature extraction, it was observed that the H&Y stages of the patients were unevenly distributed in the dataset, where each stage corresponds to a class in the dataset. A majority of the samples belonged to H&Y stage 2 (59.14%) and stage 2.5 (30.1%) and only 10.75% of the samples are stage 3, which is a large imbalance of class sizes. Since a network trained on a dataset with imbalanced classes can face problems distinguishing between different classes (An et al., 2001), this problem was addressed by generating synthetic samples of the minority class, known as Synthetic Minority Oversampling Technique (SMOTE) (Chawla et al., 2002). Table 4 shows the dataset sample distribution before and after SMOTE was performed.

**TABLE 4:** Summary of dataset classes and their sample sizes before and after synthetic sample generation.

	**Original**	***SMOTE Samples***	***After SMOTE***
Stage 1: H&Y 3	10 (10.75%)	20	*30 (21.27%)*
Stage 2: H&Y 2.5	28 (30.1%)	28	*56 (39.71%)*
Stage 3: H&Y 2	55 (59.1%)	0	*55 (39.00%)*

A peak detector was used on the output of Sensor 1 and Sensor 8 to obtain the initial contact (IC) and terminal contact (TC) magnitude and times, respectively. Subsequently, the initial double stance period and terminal double stance period was calculated using the formula established in Salarian et al. (2004).

**FIGURE 4:**
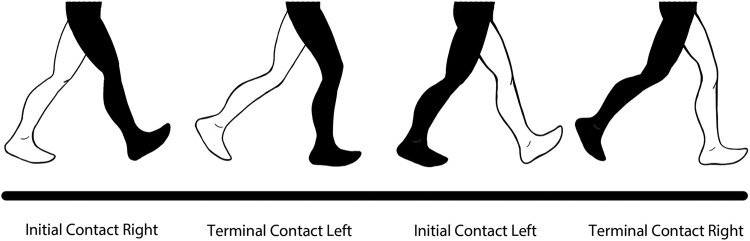
Order of Gait Cycle Events used in the validation equation.

A validation check is performed to ensure the gait parameters obtained corresponds to the correct gait event. We assume that the order of events in each gait cycle are as follows: Initial Contact of right foot (IC_*R*_), Terminal contact of left foot (TC_*L*_), Initial Contact of left foot (IC_*L*_) and Terminal Contact of the right foot (TC_*R*_), as illustrated in Figure 4.

Then, the validation equation used is (9).

(9) $$I\,C_{L}\,\left(j\right)<T\,C_{R}\,\left(j-1\right)<I\,C_{R}\,\left(j\right)<T\,C_{L}\,\left(j\right)$$

where *j* is the corresponding gait cycle iteration. Any cycle that does not meet this validation condition is excluded from analysis. Using the validated data, the following features are obtained (Eq. 10 to 13), where *j* represents the gait cycle, as shown in Table 5.

**TABLE 5:** Spatiotemporal features extracted from VGRF data.

**Feature**	**Formula**	**Eq. No.**
Initial Double Support	$I\,D\,S\,\left(j\right)=\,\frac{T\,C_{L}\,\,\left(j\right)-\,I\,C_{L}\,\left(j\right)}{G\,C\,T_{L}\,\left(j\right)}\,\times 100$	(10)
Terminal Double Support	$T\,D\,S\,\left(j\right)=\,\frac{T\,C_{R}\,\,\left(j-1\right)\,-\,I\,C_{R}\,\left(j\right)}{G\,C\,T_{L}\,\left(j\right)}\,\times 100$	(11)
Double Support	*DS*(*j*) = *IDS*(*j*) + *TDS*(*j*)	(12)
Limp	*Limp*(*j*) = |*IDS*(*j*)−*TDS*(*j*)|	(13)

### Neural Network Model for PD Diagnosis

#### Network Architecture and Training Parameters

A pattern recognition network was created using MATLAB r2017b to study the performance of the extracted gait parameters. The 34 unique input features are input into the pattern recognition network as the input layer, which consists of one hidden layer and a binary target (PD or Healthy) in the output layer. The input features are normalized to [-1,1] using MATLAB

**FIGURE 5:**
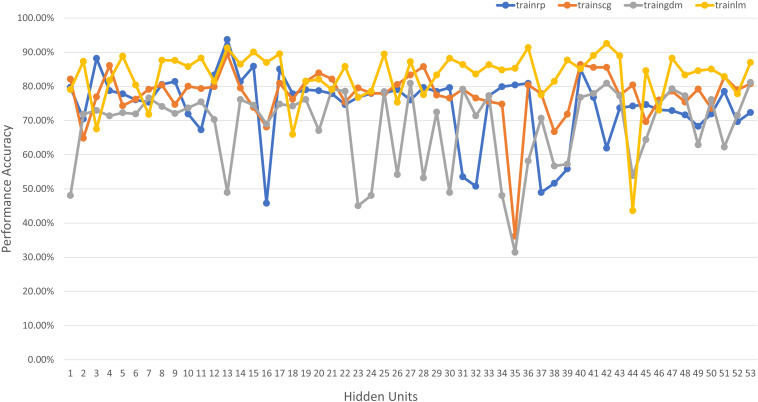
Network performance vs. hidden unit count for different training algorithms for PD diagnosis.

Neural Network Toolbox’s *mapminmax* preprocessing parameter to remove data range differences i.e., approximations between large and small data values during training to reduce classification error. The architecture of the classifier was selected iteratively based on multiple sets of training algorithm and hidden unit patterns, out of which the best was selected. Figure 5 illustrates the different training algorithms tested for each hidden unit combination, where *trainrp* is resilient backpropagation, trainlm is Levenberg-Marquardt, *traingdm* is gradient descent with momentum, and *trainscg* is scaled conjugate gradient backpropagation. The best performance occurred for the resilient backpropagation algorithm (*trainrp*) with 25 hidden neurons and was therefore chosen as the network architecture.

### Neural Network Model for PD Severity Assessment

**FIGURE 6:**
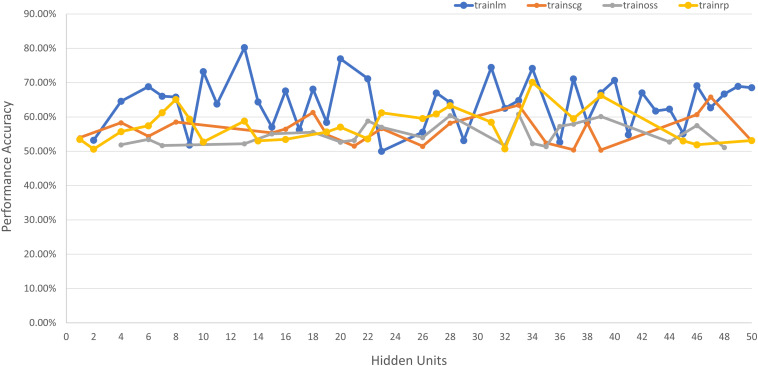
Network performance vs. hidden unit count for different training algorithms for severity assessment.

An additional pattern classification network is used to classify the patients’ disease progression based on spatiotemporal features into 3 separate classes for H&Y stages of 2, 2.5, and 3, respectively (as all the subjects in the database used fall under these three classes). The network architecture is selected based on iterative combinations of hidden units and training algorithms, which are shown in Figure 6. The tested algorithms include Levenberg-Marquardt (*trainlm*), Scaled Conjugate Gradient (*trainscg*), Gradient Descent with Momentum (*traingdm*) and Resilient Backpropagation (*trainrp)*. The best performance was achieved using 13 neurons in the single hidden layer and the Levenberg-Marquardt training algorithm (*trainlm*) and the hyperbolic tangent activation function.

## Results and Data Analysis

### Performance Analysis for PD Diagnosis

The pattern recognition network used for PD diagnosis performs well with a classification accuracy of 97.4% and a mean square error value of 0.0279, which is consistent with literature that proves a high correlation between gait variability and presence of PD (Gaenslen and Daniela, 2010), thus resulting in an accurate classification. The accuracy (ACC), error (ERR), sensitivity (SN), specificity (SP), precision (PR) and false positive rate (FPR) also indicate good results, shown in Table 6.

**TABLE 6:** The Accuracy (ACC), error (ERR), sensitivity (SN), specificity (SP), precision (PR) and false positive rate (FPR) for PD Diagnosis Classifier.

**ACC**	**ERR**	**SN**	**SP**	**PREC**	**FPR**
0.9741	0.0258	0.9770	0.9705	0.9770	0.0294

**FIGURE 7:**
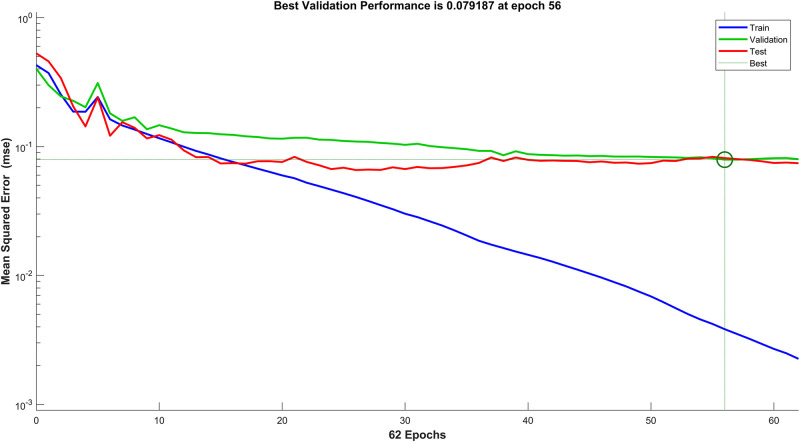
Performance against mean squared error (MSE) loss of the PD diagnosis neural network.

Figure 7 shows the performance vs. loss for the chosen architecture for PD diagnosis, where the horizontal axis is the epoch count and the vertical axis depicts the Mean Squared Error (MSE) loss. It can be observed that after 62 epochs the network converges with an MSE of 0.079 on the validation and test data.

K-fold cross validation and leave-one-out cross validation were used to ensure that the network performed as desired and did not overfit the data. Table 7 shows the results of cross validation performed and network performance. It can be observed that the model still performs well on different sets of unseen data, which shows good generalization ability and minimal overfitting (Cawley and Talbot, 2010).

**TABLE 7:** Results of different cross validation methods on the PD diagnosis classifier.

**Cross validation method (k)**	**MSE performance**	**Classification accuracy**
1-fold	0.0279	97.4%
2-fold	0.1195	84.0%
5-fold	0.0403	90.9%
10-fold	0.0332	87.9%
Leave-one-out	0.0287	94.9%

### Performance Analysis for PD Severity Assessment

**FIGURE 8:**
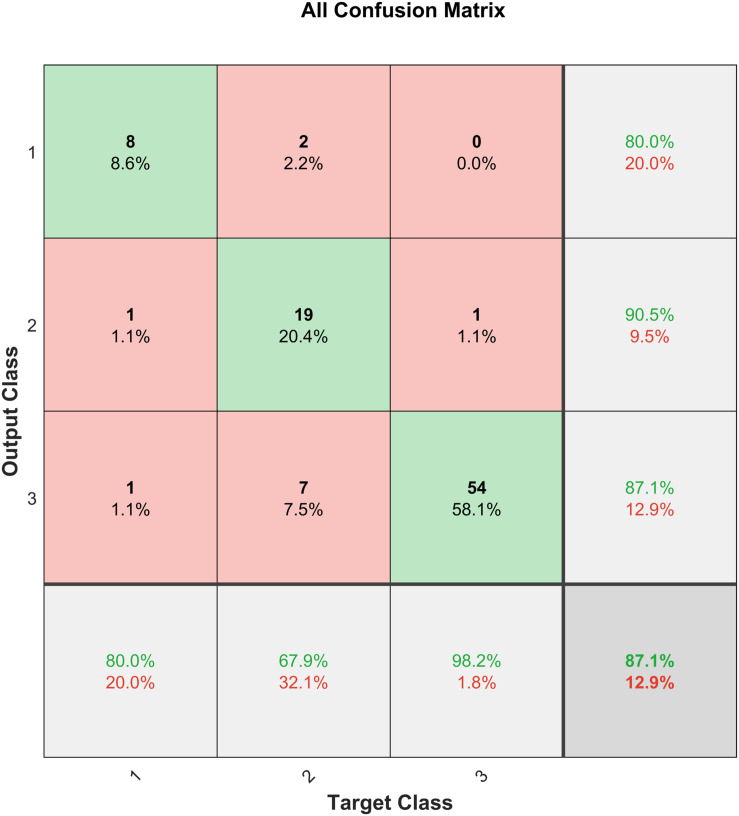
Confusion matrix for the H&Y staging classifier before SMOTE.

Performance measures are usually represented as a confusion matrix for classification problems, where the rows and columns are the predicted and target class, respectively. The diagonals depict the correctly predicted samples (True Positive (TP) or True Negative (TN) and the off-diagonal cells correspond to the incorrectly classified samples (False Positive (FP) and False Negative (FN)). Additionally, the last column on the right shows the precision (positive predictive value) and the last row shows the recall rate (sensitivity or true positive rate) and the false negative rate. The cell on the bottom right depicts the overall accuracy (Mathworks, 2019). The confusion matrix for H&Y staging classifier is shown in Figure 8.

**FIGURE 9:**
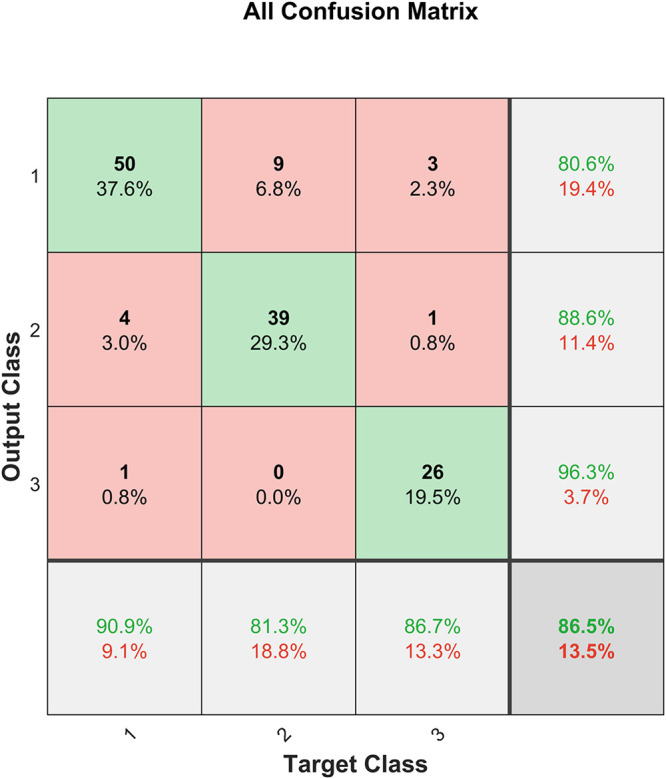
Confusion matrix for the H&Y staging classifier after SMOTE.

The H&Y staging classifier performs well, with an accuracy of 87.1%. It is also observed from the confusion matrix in Figure 8 that the H&Y classifier has a precision of 90.5% and sensitivity of 67.9% for class 2 (H&Y stage 2.5). This shows that the classifier is conservative for this class, but the opposite is true for class 3 (H&Y stage 2) for which the classifier is biased (Santos et al., 2018). This may be attributed to the bias in the dataset, where more data is available for stage 2 and 2.5 compared to stage 3, which can skew results unfairly. Figure 9 shows the confusion matrix for the same network after implementing SMOTE techniques to balance the dataset.

It can be observed from Figure 9 that after SMOTE the classifier does not exhibit an unfair bias towards any particular class, while maintaining a similar prediction accuracy.

Using the additional data obtained from SMOTE techniques, we run multiple cross validation methods for testing and error analysis, including k-fold cross validation and leave-one-out cross validation. This helps against overfitting of the data. Table 8 shows the results of the cross validation performed for PD severity assessment and the respective network performance. It is observed that the network performs consistently over multiple *k*-values, which shows good generalization (Cawley and Talbot, 2010).

**TABLE 8:** Results of different cross validation methods on the severity assessment classifier.

**Cross validation method (k)**	**MSE performance**	**Classification accuracy**
1-fold	0.0261	86.5%
5-fold	0.1063	73.06%
10-fold	0.0905	76.08%
15-fold	0.0873	77.3%
Leave-one-out	0.0688	87.69%

## Discussion

Good classification of PD subjects from healthy controls is achieved with an accuracy of 97.4% using input features extracted from VGRF data. Good accuracy of 87.1% was achieved in H&Y staging of patients’ disease progression based on their spatiotemporal and kinetic features. However, as shown in Figure 8, the severity assessment data is unevenly distributed with majority of the samples being in H&Y stage 2 (59.14%) and stage 2.5 (30.1%) and only 10.75% of the samples are stage 3. Therefore, the classifier is biased towards the majority sample class, and this could affect the generalization ability of the classifier. This is dealt with using SMOTE (Chawla et al., 2002), and the result is shown in Figure 9. It can be observed that the test accuracy improved due to the introduction of new samples in Class 1 (H&Y Stage 3). This resulted due to the availability of more data samples for training, validation, and testing. Furthermore, as the dataset becomes more equally represented using SMOTE, the network was able to perform better on unseen data, with a test accuracy of 76.9%, which is an improvement of 21.3%. The network also exhibits lesser bias towards any particular class, and the overall accuracy is 87.2%, which is also an improvement.

In comparison to the performance reported by (Manap et al., 2011; Lee and Lim, 2012; Perumal and Sankar, 2016; Zeng et al., 2016; Alam et al., 2017; Khoury et al., 2019), the proposed methodology in this work builds on and improves previous studies that use this VGRF database. This may be attributed to the extra analysis done to ensure the optimum network architecture was selected, and the combination of multiple features that proved successful in various past work, in addition to a new feature (Asymmetry Index) extracted using the same VGRF data. The result achieved in this work also outperforms work that requires data to be collected via multiple sensors located at different parts of the patients’ physique (Manap et al., 2011; Md Tahir and Manap, 2012; Klucken et al., 2013; Abdulhay et al., 2018). This is an added advantage for the proposed method, that it is able to prospectively diagnose PD with good accuracy using minimal data that may be obtained in a non-intrusive way via foot-worn sensors alone, for example embedded in subjects’ shoes.

However, in 2018 (Aşuroğlu et al., 2018) proposed a Hybrid Machine Learning (ML) model (Locally Weighted Random Forest) and achieved a classification accuracy of 99%. Though our classifier does not outperform this, it is worth noting that the work presented in this paper achieves a relatively close result using a comparatively less complicated network architecture. The classifier achieves an accuracy of 97.4% with a lightweight architecture and results that surpass or are competent with those achieved by complex methods such as Support Vector Machines (SVM) and Hybrid ML models.

Furthermore, the proposed method also carries out severity assessment corresponding to the H&Y scale using features extracted VGRF data only, which is a scarcely researched area, as most researchers use additional information apart from VGRF, such as speech data to quantify disease progression (Salarian et al., 2004; Benmalek et al., 2015; Schlachetzki et al., 2017; Grover et al., 2018; Nilashi et al., 2018). However, there are studies that have performed better in terms of severity assessment using gyroscope and accelerometer data (Klucken et al., 2013) using complex models, but to the best of our knowledge, no studies use wearable sensor based VGRF data for this purpose. The proposed severity assessment method achieves a high accuracy in predicting patients’ H&Y scores using VGRF data. This was the expected outcome as spatiotemporal gait features show good correlation with H&Y stages (Schlachetzki et al., 2017).

Furthermore, additional data generated from this study would also be useful in overcoming the bias exhibited by the classifier towards earlier H&Y stages, as the current dataset is small and prone to overfitting, and exhibits a large imbalance in the distribution class samples.

We also successfully demonstrate the feasibility of the proposed novel approach of assessing PD severity using standalone VGRF data based on the H&Y scale with an accuracy of 86.5% after SMOTE. Cross Validation methods also resulted in promising values of 76.08% for 10-fold cross validation and 87.69% for leave-one-out cross validation. This shows that the classifier is able to generalize without overfitting or exhibiting bias towards any particular class.

Apart from its application in assisting clinicians in improving the accuracy of their assessments, this framework can also be implemented as a computational layer over smart wearables like smart insole shoes that can collect VGRF data, so that disease progression monitoring can be carried out remotely without requiring frequent clinic visits. This is possible as this is a lightweight ML architecture that does not require high processing power, thus making an integration with wearable sensors feasible. By reducing the frequency of clinical visits, this framework improves patients’ and their caregiver’s quality of life. As our framework operates on lightweight architecture and can be implemented online, it poses many benefits of portability, ease of use and functionality as opposed to non-portable gait analysis systems.

It is worth noting that this study is limited to the dataset size of control and PD subjects, and only investigates ground reaction forces in the vertical direction, as the dataset contains historical data that does not capture other directions of ground reaction forces. Although our study successfully showed that PD diagnosis and severity assessment can be done to a reasonable extent with VGRF only, further study is encouraged with a bigger sample size to investigate aspects such as predictive gait pattern tracking and integration with smart insole shoes to achieve a positive societal impact in the monitoring of movement disorders in the future.

## Conclusion

A holistic, non-intrusive system is proposed for PD diagnosis and severity assessment using VGRF data from an online database collected from 166 subjects (93 PD and 73 healthy control subjects). A high classification accuracy of 97.4% is achieved using a simple ANN architecture, which confirms and extends the results of previous studies in this field that employ complex models to perform classification. Severity assessment is accurately carried out on the H&Y scale to an accuracy of 87.1% using features extracted only from VGRF data. The system as a whole is a simple and effective approach to PD diagnosis and severity assessment using only VGRF data obtained which is non-intrusive.

## Data Availability Statement

Publicly available datasets were analyzed in this study. This data can be found here: https://physionet.org/content/gaitpdb/1.0.0/.

## Author Contributions

SV and AG conceived and designed the study. SV analyzed the data. SV and AG drafted the manuscript. SV, AG, SA, and DG edited the manuscript. All authors read, commented, approved the final manuscript, contributed to the article, and approved the submitted version.

## Conflict of Interest

The authors declare that the research was conducted in the absence of any commercial or financial relationships that could be construed as a potential conflict of interest.

## References

[B1] Abdulhay E. Arunkumar N. Narasimhan K. Vellaiappan E. Venkatraman V. (2018). Gait and tremor investigation using machine learning techniques for the diagnosis of Parkinson disease. *Futur. Gener. Comput. Syst.* 83 366–373. 10.1016/j.future.2018.02.009

[B2] Alam M. N. Garg A. Munia T. T. K. Fazel-Rezai R. Tavakolian K. (2017). Vertical ground reaction force marker for Parkinson’s disease. *PLoS One* 12:175951. 10.1371/journal.pone.0175951 28493868 PMC5426596

[B3] An A. Cercone N. Huang X. (2001). “A case study for learning from imbalanced data sets,” in *Proceedings of the 14th Biennial Conference of the Canadian Society for Computational Studies of Intelligence, AI 2001: Advances in Artificial Intelligence*, Ottawa, 1–15.

[B4] Aşuroğlu T. Açıcı K. Erdaş ÇB. Toprak M. K. Erdem H. Oğul H. (2018). Parkinson’s disease monitoring from gait analysis via foot-worn sensors. *Biocybern. Biomed. Eng.* 38 760–772. 10.1016/j.bbe.2018.06.002

[B5] Benmalek E. Elmhamdi J. Jilbab A. December N. (2015). UPDRS tracking using linear regression and neural network for Parkinson’s disease prediction. *Int. J. Emerg. Trends Technol. Comput. Sci* 4 189–193.

[B6] Bhosale T. Kudale H. Kumthekar V. Garude S. Dhumal P. (2016). Gait analysis using wearable sensors. *Int. Conf. Energy Syst. Appl. ICESA* 2015 267–269.

[B7] Cawley G. C. Talbot N. L. C. (2010). On over-fitting in model selection and subsequent selection bias in performance evaluation. *J. Mach. Learn. Res.* 11 2079–2107.

[B8] Chawla N. V. Bowyer K. W. Hall L. O. Kegelmeyer W. P. (2002). SMOTE: synthetic minority over-sampling technique. *J. Artif. Intell. Res.* 16 321–357. 10.1613/jair.953

[B9] Gaenslen A. Daniela B. (2010). Early diagnosis of Parkinson’s disease. *Int. Rev. Neurobiol.* 90 81–92. 20692495 10.1016/S0074-7742(10)90006-8

[B10] Goetz C. G. Tilley B. C. Shaftman S. R. Stebbins G. T. Fahn S. Martinez-Martin P. (2008). Movement disorder society-sponsored revision of the unified Parkinson’s Disease rating scale (MDS-UPDRS): scale presentation and clinimetric testing results. *Mov. Disord.* 23 2129–2170. 10.1002/mds.22340 19025984

[B11] Goldberger A. Amaral L. Glass L. Hausdorff J. Ivanov P. C. Mark R. (2000). PhysioBank, physiotoolkit, and physionet: components of a new research resource for complex physiologic signals. *Circulation [Online]* 101 e215–e220. 10.1161/01.cir.101.23.e215 10851218

[B12] Grover S. Bhartia S. Akshama A. Y. Seeja K. R. (2018). Predicting severity of Parkinson’s disease using deep learning. *Proc. Comput. Sci.* 132 1788–1794. 10.1016/j.procs.2018.05.154

[B13] Khoury N. Attal F. Amirat Y. Oukhellou L. Mohammed S. (2019). Data-driven based approach to Aid Parkinson’s disease diagnosis. *Sensors (Basel Swit.)* 19:242. 10.3390/s19020242 30634600 PMC6359125

[B14] Klucken J. Barth J. Kugler P. Schlachetzki J. Henze T. (2013). Unbiased and mobile gait analysis detects motor impairment in Parkinson’s Disease. *PLoS One* 8:e56956. 10.1371/journal.pone.0056956 23431395 PMC3576377

[B15] Koozekanani S. H. Balmaseda M. T. Jr. Fatehi M. T. Lowney E. D. (1987). Ground reaction forces during ambulation in parkinsonism: pilot study. *PubMed. Cent.* 68 28–30. 3800620

[B16] Lee S. H. Lim J. S. (2012). Parkinson’s disease classification using gait characteristics and wavelet-based feature extraction. *Expert Syst. Appl* 39 7338–7344. 10.1016/j.eswa.2012.01.084

[B17] Manap H. H. Tahir N. M. D. Yassin A. I. M. (2011). Statistical analysis of Parkinson disease gait classification using artificial neural network. *IEEE Int. Symp. Signal Process. Inf. Technol. ISSPIT* 2011 60–65.

[B18] Mathworks (2019). *DeepLearning Toolbox Documentation (R2019a), Mathworks.* Available online at: https://www.mathworks.com/help/deeplearning/ref/plotconfusion.html (accessed December 11, 2019).

[B19] Md Tahir N. Manap H. H. (2012). Parkinson Disease gait classification based on machine learning approach.pdf. *J. Appl. Sci.* 12 180–185. 10.3923/jas.2012.180.185

[B20] Nadeau S. (2014). Understanding spatial and temporal gait asymmetries in individuals post stroke. *Int. J. Phys. Med. Rehabil.* 2:201.

[B21] Nilashi M. Ibrahim O. Ahmadi H. Shahmoradi L. Farahmand M. (2018). A hybrid intelligent system for the prediction of Parkinson’s Disease progression using machine learning techniques. *Biocybern. Biomed. Eng.* 38 1–15. 10.1016/j.bbe.2017.09.002

[B22] Parkinson’s Foundation (2019). *Understanding Parkinson’s, 200 SE 1st Street, Ste 800, Miami, FL 33131, USA.* Available online at: https://www.parkinson.org/understanding-parkinsons (accessed November 23, 2019).

[B23] Perumal S. V. Sankar R. (2016). Gait and tremor assessment for patients with Parkinson’s disease using wearable sensors. *ICT Express* 2 168–174. 10.1016/j.icte.2016.10.005

[B24] Salarian A. Russmann H. Vingerhoets F. J. G. Dehollain C. Blanc Y. Burkhard P. R. (2004). Gait assessment in Parkinson’s Disease: toward an ambulatory system for long-term monitoring. *IEEE Trans. Biomed. Eng.* 51 1434–1443. 10.1109/tbme.2004.827933 15311830

[B25] Santos M. S. Soares J. P. Abreu P. H. Araujo H. Santos J. (2018). Cross-validation for imbalanced datasets: Avoiding overoptimistic and overfitting approaches [Research Frontier]. *IEEE Comput. Intell. Mag.* 13 59–76. 10.1109/MCI.2018.2866730

[B26] Schlachetzki J. C. M. Barth J. Marxreiter F. Gossler J. Kohl Z. Reinfelder S. (2017). Wearable sensors objectively measure gait parameters in Parkinson’s disease. *PLoS One* 12:e0183989. 10.1371/journal.pone.0183989 29020012 PMC5636070

[B27] Stöckel T. Jacksteit R. Behrens M. Skripitz R. Bader R. Mau-Moeller A. (2015). The mental representation of the human gait in young and older adults. *Front. Psychol.* 6:943. 10.3389/fpsyg.2015.00943 PMC4500916 26236249

[B28] Tadano S. Takeda R. Miyagawa H. (2013). Three dimensional gait analysis using wearable acceleration and gyro sensors based on quaternion calculations. *Sensors (Switzerland)* 13 9321–9343. 10.3390/s130709321 23877128 PMC3758651

[B29] Zeng W. Liu F. Wang Q. Wang Y. Ma L. Zhang Y. (2016). Parkinson’s disease classification using gait analysis via deterministic learning. *Neurosci. Lett.* 633 268–278. 10.1016/j.neulet.2016.09.043 27693437

